# Comparative Proteomic Analysis of Differential Proteins in Response to Aqueous Extract of *Quercus infectoria* Gall in Methicillin-Resistant *Staphylococcus aureus*

**DOI:** 10.1155/2016/4029172

**Published:** 2016-09-05

**Authors:** Radhiah Khairon, Noraziah Mohamad Zin, Mariati Abdul Rahman, Dayang Fredalina Basri

**Affiliations:** ^1^School of Diagnostic & Applied Health Sciences, Faculty of Health Sciences, Universiti Kebangsaan Malaysia, Jalan Raja Muda Abdul Aziz, 50300 Kuala Lumpur, Malaysia; ^2^Department of Clinical Oral Biology, Faculty of Dentistry, Universiti Kebangsaan Malaysia, Jalan Raja Muda Abdul Aziz, 50300 Kuala Lumpur, Malaysia

## Abstract

The aim of this study is to analyze the differential proteins in MRSA ATCC 33591 treated with aqueous extract from* Q. infectoria* gall. Protein extracts were obtained from MRSA cells by sonication and were separated by 2D polyacrylamide gels. Protein spots of interest were extracted from the gels and identified using LC-ESI-QTOF MS. The concentration of* Q. infectoria* extract used for 2D-gel electrophoresis was subinhibitory concentration. Minimum inhibitory concentration (MIC) value of the extract against MRSA was 19.50 *μ*g/mL with bacteriostatic action at 1x MIC from time-kill assay. However, the extract exhibited dose-dependent manner and was bactericidal at 4x MIC with more than 3 log_10_ CFU/mL reduction at 4 h. 2D-GE map showed that 18 protein spots were upregulated and another six were downregulated more than twofold (*p* < 0.05) after treatment with subinhibitory concentration. Out of six proteins being downregulated, four proteins were identified as ferritin and catalase, branched-chain alpha-keto acid dehydrogenase subunit E2, and succinyl-CoA ligase [ADP-forming] subunit beta. Seven upregulated proteins which have been successfully identified were 3-hydroxyacyl-CoA dehydrogenase, NAD binding domain protein, formate C-acetyltransferase, 3-hydroxyacyl-[acyl-carrier-protein] dehydratase FabZ, NAD dependent epimerase/dehydratase family protein, and phosphopantothenoyl cysteine decarboxylase. It is postulated that the main mechanism of aqueous extract from gall of* Q. infectoria* was most likely involved in energy metabolism and protein stress.

## 1. Introduction

Increasing prevalence and development of resistance to existing antibacterial agent by the bacteria have become a major threat to human health for centuries.* Staphylococcus aureus* is a gram-positive pathogen which rapidly developed into methicillin-resistant* Staphylococcus aureus* (MRSA) not long after antibiotic was introduced [1]. MRSA caused both nosocomial acquired (HA) MRSA and community acquired (CA) MRSA [2]. Glycopeptide derivative such as vancomycin is an agent of last resort for the treatment of this pathogen despite recent resistance of MRSA towards this drug [3]. Combating bacterial infection never ends, and along with the development of resistance to current antibiotic, an alternative phytotherapeutic agent is needed to overcome these problems with MRSA infection.

Phytomedicine has been widely used since ancient time and became important source of pharmaceutical agent. In recent year, the world market in herbal industry is getting huge and increasing in demand. The World Bank recorded an increase in trade for herbal medicine, botanical medicine product, and raw materials with yearly growth rate between 5% and 15% [4]. Most of natural plant compounds were developed as both antimicrobial [5] and anticancer [6]. Natural products gain interest among many researchers due to their chemotherapeutical properties exerted by their active metabolite against various infections [7].

*Quercus infectoria* or “manjakani” is small oak tree indigenous to Asia minor. Malaysian women, commonly among the Malays, use the herb traditionally for postpartum care. The aqueous extract of galls from* Q. infectoria* has skin whitening effect and antioxidant property by inhibiting the superoxide and 1,1-diphenyl-2-picrylhydrazyl (DPPH) radical scavenging activities and tyrosine activities [8]. The* Q. infectoria* aqueous extract was reported to have high hydrolysable tannin content which inhibits the lethality of the Najakaouthia (Thai cobra) venom [9]. The extract was also highly capable as an antimicrobial agent against* Escherichia coli* O157:H7 [10].

Proteomics is the study of the structure and function of proteins in biological system to give a better understanding of the complex nature of the organism. Nowadays, the study of proteomics is also applied as a tool to study adaption, regulation protein, or global response of the bacteria to the environment, including against antibiotic stress [11–13]. A few studies employed proteomic approach to elucidate the effectiveness of natural product as antibacterial agent [14–17]. However there is no study with regard to the protein expression profile of MRSA on exposure to aqueous extract of* Q. infectoria* gall despite extensive studies on the anti-MRSA activity of this plant. Proteomics provide useful information that can be used to analyze differences in protein expression between untreated bacterial cells and those treated with inhibitory concentrations of aqueous extract of* Q. infectoria* gall. Thus, investigation of the proteins expressed in MRSA exposed to the aqueous extract of* Q. infectoria* galls could potentially help to elucidate the response of MRSA in the presence of the tested agent and perhaps can unravel its mechanism of anti-MRSA through identification of the protein markers.

## 2. Materials and Methods

### 2.1. Preparation of* Q. infectoria* Gall Extract

The galls were crushed to small pieces using pestle and mortar and powdered in an electric grinder. Then, the powdered specimen was dissolved in distilled water for 24 hr at 45°C and centrifuged at 3000 ×g at 4°C. The supernatant was then filtered and the whole process was repeated using the remaining residue with 300 mL distilled water. The filtrates were combined and freeze-dried at −50°C under vacuum for 12 hr to produce a fine crystal-like crude aqueous extract. The extracts were stored in air-tight jars at 4°C until further use.

### 2.2. Bacterial Strains

Only one bacterial strain was used in this study, namely, MRSA ATCC 33591. The bacterial isolate was maintained and grown in Mueller-Hinton Broth (Merck) for 18–20 hr at 37°C.

### 2.3. Determination of Minimal Inhibitory Concentration (MIC)

The MIC values of the extract were determined by microbroth dilution method according to Clinical Laboratory Standardization Institute [18] guideline with slight modifications. The aqueous extract was dissolved in sterile distilled water to a final concentration of 20 mg/mL and filtered through a 0.45 *μ*m membrane filter (Whatman, USA). Twofold serial dilution of the extract was prepared in a 96-well microtiter plate. An exponential phase of MRSA growth was diluted to obtain 10^8^ CFU/mL in Mueller-Hinton broth (MHB) which originally contained 5 × 10^5^ CFU/mL bacterial culture incubated at 37°C for 16–20 hr. The MIC value was defined as the lowest concentration of the extract that inhibits the bacterial growth.

### 2.4. Time-Kill Assay

An overnight culture of MRSA was inoculated to 10 mL of MHB, each containing an aqueous extract at concentrations 0.5x, 1x, 2x, and 4x MIC to a final cell density of approximately 5 × 10^5^ CFU/mL. The bacterial culture in the presence of vancomycin and the bacteria in MHB only were, respectively, used as positive control and growth control. A 10 *μ*L aliquot of each dilution was plated out on Mueller-Hinton agar at 0, 2, 4, 6, 8, and 24 hr. After incubating for 24 hr at 37°C, viable colonies of MRSA were counted and a graph of log CFU/mL against time was plotted.

### 2.5. SDS-PAGE

The protein samples of 100 *μ*g were suspended in sample buffer which comprised 7 M urea, 2 M tiourea, 2% CHAPS and 0.5% IPG buffer, and 0.05% (w/v) bromophenol blue. The protein samples were applied on acrylamide gels and separated by gel electrophoresis apparatus (BioRad). Proteins were separated based on their molecular weight by electrophoresis at 100 V through a stacking gel (4% acrylamide) for 10 min and a separating gel (12.5% acrylamide) at 150 V for 1 hr. The gels were then stained by overnight incubation with gentle agitation in staining solution (0.3% (w/v) Coomassie Brilliant Blue R-250 in 45% (v/v) methanol, 40% (v/v) acetic acid). Prestained protein ladder (Invitrogen) was used as SDS-PAGE molecular weight marker.

### 2.6. Preparation of 2-DE Gel Samples

MRSA 33591 culture was grown overnight with constant shaking in MHB in the presence or absence of the extract at subinhibitory concentration of 9.75 *μ*g/mL with three biological triplicates. The suspension was centrifuged at 4,000 ×g for 30 min at 4°C and was then washed by resuspending in 10 mL phosphate buffer.

The bacteria pellet was then resuspended in cell lysis buffer (7 M urea, 2 M thiourea, 4% CHAPS, 2% IPG buffer, 40 mM dithiothreitol, and DTT) containing protease inhibitor (GE Healthcare). The mixtures were sonicated in a tip-probe sonicator (Fisher Scientific) on ice for 1 min. The procedure was repeated five times with 1 min interval on ice. The lysate was then centrifuged at 10,000 ×g at 4°C for 10 min. After precipitation with 100% ethanol, the protein was centrifuged at 10,000 ×g for 15 min at 4°C. The protein sample was then dissolved in buffer solution (7 M urea, 2 M thiourea, 4% CHAPS, 2% IPG buffer, and 40 mM DTT). The concentration of protein sample was quantified by Bradford protein assay kit (Bio-Rad, US).

### 2.7. Two-Dimensional Gel Electrophoresis (2-DE)

Protocols of 2-DE were performed according to previously described method [19] with three technical triplicates. The protein samples were separated on immobilized pH gradient (IPG) strips of 24 cm with a pH range 4–7. The IPG strips were rehydrated with 450 *μ*L IPG rehydration buffer. The samples were applied onto the acidic region of the slot in the strip. Isoelectric focusing (IEF) was performed with the Ettan IPG Phor System (GE Healthcare) according to the manufacturer's guideline at a total voltage of 60 kVh. IPG strips were equilibrated in 10 mL of SDS-equilibrium buffer (6 M urea, 75 mM Tris-HCl, 30% glycerol, 2% SDS, and 0.002% bromophenol blue) containing 50 mg DTT for 15 min at room temperature for the reduction reaction. The strip was alkylated by immersing in 10 mL of equilibration buffer containing 250 mg iodoacetamide (IAA) for 15 min and placed on 12.5% SDS-polyacrylamide gel.

The second dimension was run using EttanDalt Six (GE Healthcare) at 15°C 10 mA gel^−1^ for 1 hr, the current was then increased to 40 mA gel^−1^, and the run was continued until the bromophenol blue dye had moved towards the end of the gel. After the 2-GE run, the gels were fixed in a mixture (0.4% ethanol (v/v) and 0.1% glacial acetic acid (v/v)) and further stained using the silver staining with some modification [20].

### 2.8. Image Analysis

The gels were analyzed using ImageScanner III Lab Scan (GE Healthcare) and Progenesis software (Nonlinear Dynamics). The statistic used in this study for analysis was Anova in the Progenesis software. The software was used to detect the differences in the image of profile expression protein between the images of proteins from treated aqueous extract cell with the untreated cell proteins. The protein changes as well as similar protein pattern maps in the gels were analyzed from three independent experiments (biological replicate) and three technical replicates. Changes in protein expression spots were considered significant (*p* < 0.05) by Anova analysis only if the intensity of protein spots was more than twofold higher.

### 2.9. In-Gel Digestion and LC-ESI-QTOF MS

Protein spots were excised from silver stained 2-DE gels. Protein spots were destained using fresh washing solution (100 mM ammonium bicarbonate, 15 mM potassium ferrocyanide, and 100 mM sodium thiosulphate for 15 min on orbital shaker). The gel pieces were reduced with 10 mM DTT in 10 mM ammonium bicarbonate at 60°C for 30 min. For alkylation process, 55 mM IAA in 100 mM ammonium bicarbonate was added to the gel plugs and incubated at room temperature for 20 min in the dark. The gel pieces were rinsed three times in 50% acetonitrile (ACN) in 10 mM ammonium bicarbonate for 20 min on orbital shaker. Then, 100% ACN was added until the gel plugs become milky white after 15 min. To perform in-gel digestion of proteins, 25 *μ*L of trypsin solution (7 ng/*μ*L trypsin in 1% ACN/40 mM ammonium bicarbonate) was added to the gels and incubated at 37°C overnight. After incubation, 25 *μ*L of 30% ACN was added to keep the gels immersed throughout the digestion process. The supernatant which was the extract peptides was collected and dried for 1 hr. Peptides were desalted using ZipTip C18 (Millipore, US) after equilibration in 50% ACN in 0.1% formic acid. The extracted peptides were collected, pooled, dried by vacuum centrifuge, and kept at −20°C for further mass spectrometric analysis. The peptides were then eluted and injected into LC-ESI-QTOF MS (model G6550, Agilent US) with electrospray at flow rate of 160 nL/min to a nanocolumn (G4240-62010, analytical 75 *μ*m × 150 mm, Zorbax 300SB-C18, 5 *μ*m). A solvent gradient (solvent A: 50% ACN in water; solvent B: 70% ACN; solvent C: 70% ACN; solvent D: 5% acetonitrile) was run for 25 min.

### 2.10. Bioinformatics

After all the distinct peptides have undergone validation process, the mass spectra and sequences were analyzed using a public database (UniProtKB and National Center for Biotechnology Information) with more than two distinct peptides. Protein-protein interactions were predicted using Search Tool for the Retrieval of Interacting Genes/Proteins (STRING) database v9.0 (http://www.string-db.org/). The Swiss-Prot identifier for the* Staphylococcus aureus* genes, in “protein mode,” was used to search using the STRING database. Network analysis was set at medium stringency (STRING score = 0.4). Proteins were linked based on seven criteria: neighbourhood, gene fusion, cooccurrence, coexpression, experimental evidences, existing databases, and text mining.

## 3. Results

### 3.1. Antibacterial Activity

**Figure 1:**
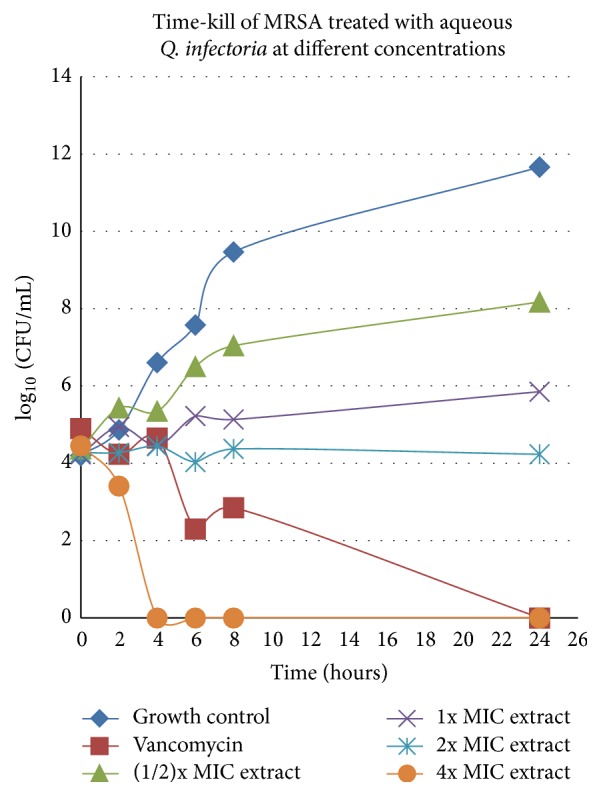
Time-kill curve of aqueous extract from* Q. infectoria* against MRSA 33591. Viability of cells growth was counted at specified hr by serial dilution. Each point represents the mean of log_10_  ± SEM (*n* = 3).

The aqueous extract showing inhibitory activity against MRSA 33591 was 19.75 *μ*g/mL, which was ten times higher than the MIC value for vancomycin. The result for the time-kill studies was presented in Figure 1, whereby the MRSA treated with subinhibitory concentration showed colony count of viable cell lower than the growth control after 24 hr incubation. Meanwhile bacteria growth was stable when treated with 1x and 2x MIC aqueous extract of* Q. infectoria* gall after 6 hr incubation. The number of viable cells after exposure to 4x MIC of extract decreased by more than 3 log_10_ CFU/mL faster at the 3rd hr compared to vancomycin after 13 hr treatment.

### 3.2. Protein Variation between Cell Cultures

**Figure 2:**
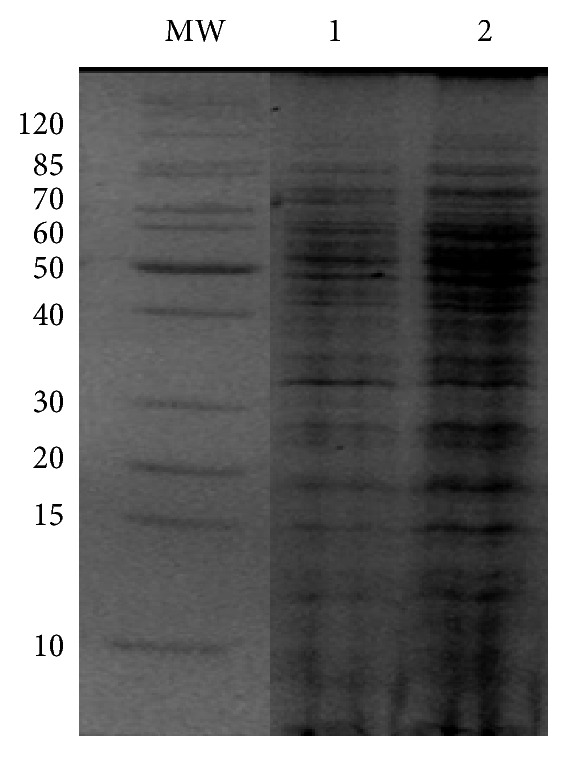
SDS-PAGE map of MRSA 33591 after being treated with aqueous extract of* Q. infectoria. *MW: protein ladder; lane number: 1 untreated MRSA; lane 2: extract-treated MRSA.

Preliminary study whether aqueous extract* Q. infectoria* gall affected staphylococcal cell protein patterns was performed by SDS-PAGE. MRSA treated with aqueous extract* Q. infectoria* gall exhibited different protein expression pattern compared to untreated MRSA in view of the level of intensity of the bands (Figure 2). 2D-GE combined with LC-ESI-QTOF MS was performed to identify the differential proteins which were affected in response to exposure with the tested extract.

**Figure 3:**
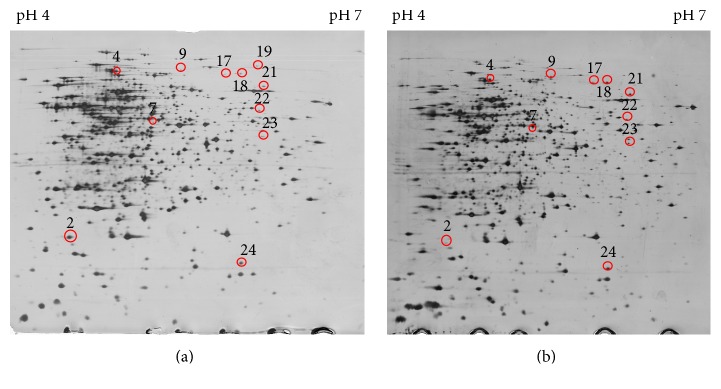
Two 2D-GE maps of the cellular MRSA proteome. (a) Protein expression profile of untreated MRSA. (b) Protein expression profile of extract treated-MRSA.

Figure 3 showed the protein mapping of treated and untreated cells observed in acidic range in which most of alterations between the two proteomes were displayed. More than 1,000 individual protein spots were resolved and visualized on silver-staining. The optimum concentration of MRSA protein for 2D-GE analysis was 100 *μ*g. Image analysis identified 24 protein spots that exhibited significant differences after treatment with the extract. There are 17 protein spots that were shown to be upregulated and another five were found to be downregulated with more than twofold increase (*p* < 0.05) (data not shown). However, only 12 of the protein spots were successfully identified; four protein spots showed downregulation whereas eight protein spots were upregulated.

### 3.3. Protein Network Analysis

**Figure 4:**
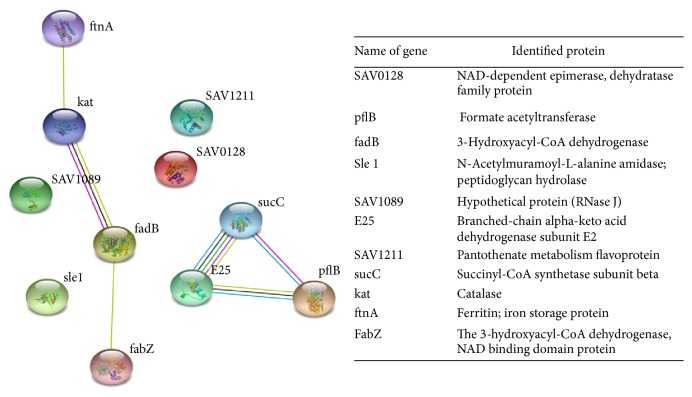
STRING interaction network analysis showed the association between differentially expressed proteins. The protein names and gene symbols used in this network are listed in the table. Higher amount of lines indicates stronger interaction between the proteins.

Predicted and identified functionally linked proteins and the determination of the potential biological process involved after treatment with aqueous extract from* Q. infectoria* gall were analyzed by STRING network analysis as shown in Figure 4. The network was presented under confident view, whereby stronger associations were represented by thicker lines or edges and proteins were represented as nodes. The interaction between the 11 proteins was tabulated (Table 1) in order to summarize the differentially expressed proteins. Four proteins were found to be linked either directly or indirectly through one or more interacting proteins, suggesting the existence of reported functional linkages. However, another group of three proteins were linked together without linkage to other groups.

## 4. Discussion

Methicillin-resistant* S. aureus* (MRSA) has become a common pathogen, particularly in the long-term care setting [21]. Thus, the treatment of MRSA infection using current antibiotic has become limited due to the emergence of multidrug-resistant strains [22]. Herbal medicine is one of the strategies to combat resistance from this infection in an effort to overcome mortality and morbidity patient problem in healthcare. Recent studies [23, 24] reported that natural product has high antimicrobial potential against MRSA. The aqueous extract from* Quercus infectoria* gall in this study showed strong anti-MRSA activity and this is in agreement with [25] that this extract showed promising capacity as new phytotherapeutic candidate against MRSA.

Time-kill assay indicated that the aqueous extract from* Q. infectoria* gall exhibited significant bactericidal activity at 4x MIC in dose-dependent manner. Bactericidal activity was defined as a reduction of more than 3 log_10_ CFU/mL at certain time compared to at 0 hr [26]. However at lower dose, the extract was bacteriostatic and subinhibitory concentration ((1/2)x MIC) of 9.75 *μ*g/mL was chosen to run 2D-GE to ensure that enough protein can be obtained in order to analyze the protein expression profile of MRSA. The presence of crude aqueous extract of* Q. infectoria* gall against MRSA remarkably interfered with the general metabolic pathway. One of the affected proteins lost was the branched-chain *α*-keto acid dehydrogenase subunit E2. It is one important component of the protein complex which is the dihydrolipoyl transacylase that catalyzes transacylation of the acyl group from E2 to coenzyme A [27]. Branched-chain *α*-keto acid dehydrogenase is an enzyme complex that catalyzes the early stages of branched-chain fatty acid production [28] and plays a key role in catabolism of branched-chain amino acids in bacteria [29]. This enzyme is important as adherence of* S. aureus* to the host eukaryotic cells for its survival in a mouse host due to its function in the membrane fluidity of* S. aureus*.

Another protein which was downregulated was succinyl-coenzyme A (CoA) ligase [ADP-forming] subunit beta which is involved in citric acid cycle for energy metabolism [30]. This enzyme reversibly catalyzes ATP-dependent ligation from succinate and CoA to form succinyl-CoA in either aerobic or anaerobic condition. During aerobic growth, succinyl-CoA ligase catalyzes the hydrolysis of succinyl-CoA using ADP and inorganic orthophosphate (*P*_*i*_) to form succinate and ATP. However, during anaerobic condition, succinyl-CoA ligase can freely interconvert ATP and succinate to form succinyl-CoA, ADP, and inorganic *P*_*i*_ for anabolic reaction as well [31].

Exposure of MRSA culture to crude extract of* Q. infectoria* gall suppressed the production of proteins that were involved in oxidative stress. These proteins were ferritin and catalase. Ferritins are the major iron storage proteins in organism and provide protection against metal toxicity and oxidative stress [32]. Ferritin was downregulated after treatment with* Q. infectoria* gall possibly due to its effect in limiting the iron environment; hence it interfered by lowering the expression of ferritin protein. Tannin content in* Q. infectoria* extract is believed to retard the growth of bacteria by inhibiting the activity of metalloenzymes in complex tannins and iron [33]. Meanwhile, catalase catalyzes hydrogen peroxide to form oxygen and water. Catalase is also known as one of the virulent factors and when its production was reduced, the bacteria cells become more susceptible to be killed by neutrophils in the host cells [34]. The proposed stress-related protein obviously reflected an adaption of the bacteria to survival under stress condition.

Cell wall in gram-positive bacteria is very important for its survival. Bacterial cell wall is composed of the cross-linked polymer peptidoglycan, also known as murein. Peptidoglycan architecture consists of a disaccharide backbone built of alternating *β*-1-4-N-acetylglucosamines and N-acetylmuramic acids [35]. Bacterial cell produces several hydrolases that specifically cleave various covalent bonds in the peptidoglycan, including* N*-acetylmuramoyl-L-alanine amidases,* N*-acetylglucosaminidases,* N*-acetylmuramidases, endopeptidases, and transglycosylases. N-acetylmuramoyl-L-alanine amidase plays important roles in the separation septum of daughter cells during cell division [36]. Hence, N-acetylmuramoyl-L-alanine amidase binds to both alpha and beta human fibrinogen and fibronectin which play a role during colonization and pathogenicity in the host tissue during infection. Previous study [37] reported that this enzyme was upregulated to activate and increase its own production in the presence of antibiotic to the bacteria and eventually cause inhibition of the bacterial growth or cell death.

Formate C-acetyltransferase or pyruvate formate-lyase was upregulated in MRSA treated with aqueous extract from* Q. infectoria* gall. This is a cytoplasmic protein involved in energy fermentation metabolism pathway in the microorganism. This enzyme was induced in anaerobic growth and limited nitrate [38, 39]. Formate C-acetyltransferase functions to catalyze pyruvate to acetyl-CoA and formate which is subsequently used for biosynthesis of protein, DNA, and RNA [40] and also as source for citric acid cycle through acetyl-CoA [41]. Formate C-acetyltransferase, NAD-dependent formate dehydrogenase, and formyltetrahydrofolate synthetase were upregulated during transcription at proteome level for the synthesis of* S. aureus* biofilm growth [42]. This enzyme was upregulated as one of the survival strategies of bacteria under stress [40].

Phosphopantothenoyl cysteine decarboxylase/phosphopantothenate-cysteine ligase is also known as acid pantothenic [43]. This is the last enzyme involved in the biosynthesis of CoA, which is a crucial substance in the biogenesis of bacterial cell membrane and functions as important cofactor in mitosis and stability of DNA in microorganism [44]. This protein was upregulated in the study probably due to compensation of other pathways to increase in the synthesis of CoA for adaptation when exposed to subinhibitory concentration of the* Q. infectoria* gall extract.

NAD-dependent epimerase/dehydratase protein is a new protein that has high similarity with UDP-glucose-4-epimerase involved in Leloir pathway [45]. The other protein that was shown to be upregulated after treatment was RNase J which is involved in maturation of RNA and regulation of posttranscription until degradation of RNA in family Firmicute of bacteria.

Other proteins that were upregulated during fatty acid metabolism are 3-hydroxyacyl-CoA dehydrogenase, NAD-binding domain, and 3-hydroxyacyl-[acyl-carrier-protein] dehydratase FabZ. The 3-hydroxyacyl-CoA dehydrogenase, NAD binding domain protein, was involved in various metabolic pathways such as beta oxidation fatty acid by catalyzing the oxidation of 3-hydroxyl coenzyme A to oxoacyl CoA and production of butanol in bacteria gram-positive organism [46]. Upregulation of this protein may be due to compensation of low level of the enzyme to tricarboxylic acid cycle [47]. In addition, another protein that was involved in the synthesis of fatty acid was 3-hydroxyacyl-[acyl-carrier-protein] dehydratase FabZ. The latter was reported to participate in the phospholipid membrane biosynthesis pathway [48].

## 5. Conclusions

In conclusion, all the data reported in this study may elucidate and give better understanding on antibacterial effect of aqueous extract of* Q. infectoria* towards MRSA through interference of various metabolism and functions of the bacterial proteome. Furthermore, recent studies suggested that aqueous extract of* Q. infectoria* galls has no toxicity effect and mortality effect in animal [49, 50]. However, due to its high tannin content which has a profound digestibility-reducing effect in human, it is ideal to be administered topically since it has the advantage of possessing the anti-inflammatory effect and wound healing activity. This information has widened the chances for alternative medicine as therapeutic agent to treat MRSA infection.

## Figures and Tables

**Table 1:** The protein and genes identified in the STRING analysis network. The list of proteins identified is differentially expressed proteins following treatment with *Q. infectoria* gall extract.

Spot number	Accession number	Proteins identified	Mascot score	Nominal mass (kDa)	Sequence coverage (%)	Number of peptides matched	Folds changes (untreated to treated)
2	FTN_SAAR	Ferritin	94	19.590	34	5	−2.4
4	D6SHZ8	Branched-chain alpha-keto acid dehydrogenase subunit E2	131.78	46.4516	23.4	8	−2.7
7	D6SHJ5	Succinyl-CoA ligase [ADP-forming] subunit beta	65.43	42.2837	13.1	5	−2.2
9	D6SDX7	Formate C-acetyltransferase	140.51	85.5525	15.7	10	2.6
10	D6SH75	Catalase	66.78	58723.3	10.2	5	−2.4
17 & 18	D6SBS5	N-Acetylmuramoyl-L-alanine amidase	28.34	69.2891	3.3	2	5.3
19	D6SDX0	3-Hydroxyacyl-CoA dehydrogenase, NAD binding domain protein	51.44	84.6729	6.3	4	2.6
21	D6SHG5	Ribonuclease J	74.12	64.6507	9.2	5	2.4
22	D6SHN0	Phosphopantothenoylcysteine decarboxylase/phosphopantothenate-cysteine ligase	31.95	44.1965	6.2	2	2.1
23	D6SDL7	NAD dependent epimerase/dehydratase family protein	27.9	37.8017	7.6	2	2.1
24	D6SEM6	3-Hydroxyacyl-[acyl-carrier-protein] dehydratase FabZ	27.31	16.3096	12.3	2	2.2

## References

[1] Crum N. F., Lee R. U., Thornton S. A. (2006). Fifteen-year study of the changing epidemiology of methicillin-resistant *Staphylococcus aureus*. *The American Journal of Medicine*.

[2] Asensio A., Cantón R., Vaqué J. (2006). Nosocomial and community-acquired meticillin-resistant *Staphylococcus aureus* infections in hospitalized patients (Spain, 1993–2003). *Journal of Hospital Infection*.

[3] Hiramatsu K., Hanaki H., Ino T., Yabuta K., Oguri T., Tenover F. C. (1997). Methicillin-resistant *Staphylococcus aureus* clinical strain with reduced vancomycin susceptibility. *Journal of Antimicrobial Chemotherapy*.

[4] WHO

[5] Brown D. G., Lister T., May-Dracka T. L. (2014). New natural products as new leads for antibacterial drug discovery. *Bioorganic & Medicinal Chemistry Letters*.

[6] Cragg G. M., Grothaus P. G., Newman D. J. (2009). Impact of natural products on developing new anti-cancer agents. *Chemical Reviews*.

[7] Dias D. A., Urban S., Roessner U. (2012). A historical overview of natural products in drug discovery. *Metabolites*.

[8] Sharififar F., Dehghan-Nudeh G., Raeiat Z., Amirheidari B., Moshrefi M., Purhemati A. (2013). Tyrosinase inhibitory activity of major fractions of *Quercus infectoria* galls. *Pharmacognosy Communications*.

[9] Pithayanukul P., Ruenraroengsak P., Bavovada R., Pakmanee N., Suttisri R., Saen-Oon S. (2005). Inhibition of *Naja kaouthia* venom activities by plant polyphenols. *Journal of Ethnopharmacology*.

[10] Voravuthikunchai S., Lortheeranuwat A., Jeeju W., Sririrak T., Phongpaichit S., Supawita T. (2004). Effective medicinal plants against enterohaemorrhagic *Escherichia coli* O157:H7. *Journal of Ethnopharmacology*.

[11] Cordwell S. J., Larsen M. R., Cole R. T., Walsh B. J. (2002). Comparative proteomics of *Staphylococcus aureus* and the response of methicillin-resistant and methicillin-sensitive strains to Triton X-100. *Microbiology*.

[12] Kohler C., Wolff S., Albrecht D. (2005). Proteome analyses of *Staphylococcus aureus* in growing and non-growing cells: a physiological approach. *International Journal of Medical Microbiology*.

[13] Van Oudenhove L., Devreese B. (2013). A review on recent developments in mass spectrometry instrumentation and quantitative tools advancing bacterial proteomics. *Applied Microbiology and Biotechnology*.

[14] Dosselli R., Millioni R., Puricelli L. (2012). Molecular targets of antimicrobial photodynamic therapy identified by a proteomic approach. *Journal of Proteomics*.

[15] Jenkins R., Burton N., Cooper R. (2011). Manuka honey inhibits cell division in methicillin-resistant *Staphylococcus aureus*. *Journal of Antimicrobial Chemotherapy*.

[16] Packer J. M., Irish J., Herbert B. R. (2012). Specific non-peroxide antibacterial effect of manuka honey on the *Staphylococcus aureus* proteome. *International Journal of Antimicrobial Agents*.

[17] Sianglum W., Srimanote P., Wonglumsom W., Kittiniyom K., Voravuthikunchai S. P. (2011). Proteome analyses of cellular proteins in methicillin-resistant *Staphylococcus aureus* treated with rhodomyrtone, a novel antibiotic candidate. *PLoS ONE*.

[18] Clinical and Laboratory Standards Institute (2005). Performance standards for antimicrobial susceptibility testing. *Fifteenth Informational Supplement*.

[19] Basri D. F., Aik L. S., Khairon R., Rahman M. A. (2013). 2-D gel electrophoresis map of methicillinresistant *Staphylococcus aureus* treated with *Quercus infectoria* gall extract. *American Journal of Biochemistry and Biotechnology*.

[20] Chevallet M., Luche S., Rabilloud T. (2006). Silver staining of proteins in polyacrylamide gels. *Nature Protocols*.

[21] Davies J., Davies D. (2010). Origins and evolution of antibiotic resistance. *Microbiology and Molecular Biology Reviews*.

[22] Livermore D. M. (2000). Antibiotic resistance in staphylococci. *International Journal of Antimicrobial Agents*.

[23] Yu L., Xiang H., Fan J. (2008). Global transcriptional response of *Staphylococcus aureus* to Rhein, a natural plant product. *Journal of Biotechnology*.

[24] Doyle M., Feuerbaum E.-A., Fox K. R., Hinds J., Thurston D. E., Taylor P. W. (2009). Response of *Staphylococcus aureus* to subinhibitory concentrations of a sequence-selective, DNA minor groove cross-linking pyrrolobenzodiazepine dimer. *Journal of Antimicrobial Chemotherapy*.

[25] Basri D. F., Fan S. H. (2005). The potential of aqueous and acetone extracts of galls of *Quercus infectoria* as antibacterial agents. *Indian Journal of Pharmacology*.

[26] Eloff J. N. (1998). A sensitive and quick microplate method to determine the minimal inhibitory concentration of plant extracts for bacteria. *Planta Medica*.

[27] Li L., Thipyapong P., Breeden D. C., Steffens J. C. (2003). Overexpression of a bacterial branched-chain *α*-keto acid dehydrogenase complex in *Arabidopsis* results in accumulation of branched-chain acyl-CoAs and alteration of free amino acid composition in seeds. *Plant Science*.

[28] Singh V. K., Hattangady D. S., Giotis E. S. (2008). Insertional inactivation of branched-chain *α*-keto acid dehydrogenase in *Staphylococcus aureus* leads to decreased branched-chain membrane fatty acid content and increased susceptibility to certain stresses. *Applied and Environmental Microbiology*.

[29] Lowe P. N., Hodgson J. A., Perham R. N. (1983). Dual role of a single multienzyme complex in the oxidative decarboxylation of pyruvate and branched-chain 2-oxo acids in *Bacillus subtilis*. *Biochemical Journal*.

[30] Kapatral V., Bina X., Chakrabarty A. M. (2000). Succinyl coenzyme A synthetase of *Pseudomonas aeruginosa* with a broad specificity for nucleoside triphosphate (NTP) synthesis modulates specificity for NTP synthesis by the 12-kilodalton form of nucleoside diphosphate kinase. *Journal of Bacteriology*.

[31] Park S.-J., Chao G., Gunsalus R. P. (1997). Aerobic regulation of the sucABCD genes of *Escherichia coli*, which encode *α*-ketoglutarate dehydrogenase and succinyl coenzyme A synthetase: roles of ArcA, Fnr, and the upstream sdhCDAB promoter. *Journal of Bacteriology*.

[32] Andrews S. C. (1998). Iron storage in bacteria. *Advances in Microbial Physiology*.

[33] Chung K.-T., Lu Z., Chou M. W. (1998). Mechanism of inhibition of tannic acid and related compounds on the growth of intestinal bacteria. *Food and Chemical Toxicology*.

[34] Mandell G. L. (1975). Catalase, superoxide dismutase, and virulence of *Staphylococcus aureus*. In vitro and in vivo studies with emphasis on staphylococcal-leukocyte interaction. *The Journal of Clinical Investigation*.

[35] Chapot-Chartier M. P., Kulakauskas S. (2014). Cell structure and function in lactic acid in bacteria. *Microbial Cell Factories*.

[36] Fischetti V. A. (2005). Bacteriophage lytic enzymes: novel anti-infectives. *Trends in Microbiology*.

[37] Heidrich C., Templin M. F., Ursinus A. (2001). Involvement of *N*-acetylmuramyl-l-alanine amidases in cell separation and antibiotic-induced autolysis of *Escherichia coli*. *Molecular Microbiology*.

[38] Fuchs S., Pané-Farré J., Kohler C., Hecker M., Engelmann S. (2007). Anaerobic gene expression in *Staphylococcus aureus*. *Journal of Bacteriology*.

[39] Rasmussen L. J., Moller P. L., Atlung T. (1991). Carbon metabolism regulates expression of the pfl (pyruvate formate-lyase) gene in *Escherichia coli*. *Journal of Bacteriology*.

[40] Leibig M., Liebeke M., Mader D., Lalk M., Peschel A., Götz F. (2011). Pyruvate formate lyase acts as a formate supplier for metabolic processes during anaerobiosis in *Staphylococcus aureus*. *Journal of Bacteriology*.

[41] Buckel W., Golding B. T. (2006). Radical enzymes in anaerobes. *Annual Review of Microbiology*.

[42] Resch A., Leicht S., Saric M. (2006). Comparative proteome analysis of *Staphylococcus aureus* biofilm and planktonic cells and correlation with transcriptome profiling. *Proteomics*.

[43] Kupke T. (2002). Molecular characterization of the 4′-phosphopantothenoylcysteine synthetase domain of bacterial Dfp flavoproteins. *The Journal of Biological Chemistry*.

[44] Nakamura T., Pluskal T., Nakaseko Y., Yanagida M. (2012). Impaired coenzyme A synthesis in fission yeast causes defective mitosis, quiescence-exit failure, histone hypoacetylation and fragile DNA. *Open Biology*.

[45] Simeonova D. D., Susnea I., Moise A., Schnik B., Przybylski M. (2009). ‘Unknown genome’ proteomics: a new NAD(P)-dependent epimerase/dehydratase revealed by N-terminal sequencing, inverted PCR, and high resolution mass spectrometry. *Molecular & Cellular Proteomics*.

[46] Clark D. P., Cronan J. E. (1996). Two carbon compounds and fatty acids as carbon sources. *Escherichia coli and Salmonella: Cellular and Molecular Biology*.

[47] Boynton Z. L., Bennett G. N., Rudolph F. B. (1996). Cloning, sequencing, and expression of clustered genes encoding beta hydroxybutyryl-coenzyme A (CoA) dehydrogenase, crotonase, and butyryl-CoA dehydrogenase from *Clostridium acetobutylicum* ATCC 824. *Journal of Bacteriology*.

[48] Schiebel J., Chang A., Lu H., Baxter M. V., Tonge P. J., Kisker C. (2012). *Staphylococcus aureus* FabI: inhibition, substrate recognition, and potential implications for in vivo essentiality. *Structure*.

[49] Iminjan M., Amat N., Li X.-H., Upur H., Ahmat D., He B. (2014). Investigation into the toxicity of traditional uyghur medicine quercus infectoria galls water extract. *PLoS ONE*.

[50] Hasmah A., Nurazila Z., Chow C. Y., Rina R., Rafiquzzaman M. (2010). Cytotoxic effects of *Quercus infectoria* extracts towards cervical (Hela) and Ovarian (Caov-3) cancer cell lines. *Health and the Environment Journal*.

